# Correction to “O-GlcNAc Modification of α‑Synuclein Can Alter Monomer Dynamics to Control Aggregation Kinetics”

**DOI:** 10.1021/acschemneuro.5c00965

**Published:** 2025-12-21

**Authors:** Kasun Gamage, Binyou Wang, Eldon R Hard, Thong Van, Ana Galesic, George R Phillips, Matthew Pratt, Lisa J. Lapidus

A grant number was missing in the Acknowledgments section (MCB 2210228). Please see corrected section.

